# Successful Two-Stage Exchange Arthroplasty for Periprosthetic Infection Following Total Knee Arthroplasty: The Impact of Timing on Eradication of Infection

**DOI:** 10.7150/ijms.47655

**Published:** 2021-01-01

**Authors:** Ines Vielgut, Gerold Schwantzer, Andreas Leithner, Patrick Sadoghi, Uldis Berzins, Mathias Glehr

**Affiliations:** 1 Department of Orthopaedics and Trauma, Medical University of Graz, Graz, Austria; 2 Institute for Medical Informatics, Statistics and Documentation, Medical University of Graz, Graz, Austria

**Keywords:** Total knee arthroplasty, Prosthetic knee joint infection, Antibiotic-augmented joint spacer, Two-stage revision procedure

## Abstract

**Background:** Periprosthetic joint infection (PJI) represents a serious complication following total knee arthroplasty. In the setting of chronic infections, the two-staged approach has traditionally been the preferred treatment method. The aim of this study was to determine the optimal period of rest between the first and second stage. Furthermore, we analyzed potentially outcome-relevant parameters, such as general and local conditions and the presence of difficult-to-treat or unidentified microorganisms, with regard to their impact on successful treatment of PJI.

**Patients and Methods:** We performed a retrospective analysis of prospectively collected data for all patients treated for PJI at our institution. Seventy-seven patients who had undergone two-stage revision arthroplasty for PJI of the knee were included into the study. Antibiotic-loaded cement spacers were used for all patients.

**Results:** After a median follow-up time of 24.5 months, infection had reoccurred in 14 (18.7%) patients. A prolonged spacer-retention period of more than 83 days was related to a significantly higher proportion of reinfections. Furthermore, significant compromising local conditions of the prosthetic tissue and surrounding skin, as well as repeated spacer-exchanges between first- and second-stage surgery, negatively influenced the outcome. Neither the patients' age nor gender exerted a significant influence on the outcome regarding reinfection rates for patients' age or gender.

**Conclusions:** We observed the best outcome regarding infection control in patients who had undergone second-stage surgery within 12 weeks after first-stage surgery. Nearly 90% of these patients stayed free from infection until the final follow-up. An increased number of performed spacer-exchanges and a bad local extremity grade also had a negative impact on the outcome.

## Introduction

Despite continuous innovations and improvements in knee arthroplasty, the occurrence of prosthetic joint infections (PJI) has not decreased. Although the overall PJI incidence following total knee arthroplasty is low (1-2%), PJI represents a serious complication with potentially devastating effects to the patients 1-3. Two-stage exchange arthroplasty has been the procedure of choice for chronic infections. Although this procedure has consistently demonstrated infection control rates of 80-90%, there are still cases where this method fails 3. There are several possible explanations. First, there are marked differences regarding individual circumstances (resistant or difficult to treat microorganisms, worse local and general conditions). Second, the treatment protocol for two-stage exchange arthroplasty may vary depending on geographic and/or empiric experiences of the treating department 2-7. For example, to our knowledge there are no specific recommendations concerning the length of joint-spacer retention 1, 3, 8-9. We hypothesized that the duration of spacer-retention plays an essential role for treatment success.

In order to determine the optimal period of rest between the first and second stage, we analyzed the data of 77 consecutive patients who had undergone two-stage exchange arthroplasty for prosthetic knee joint infections with regard to successful infection control. Furthermore, we analyzed potentially outcome-relevant parameters (general and local conditions, difficult-to-treat microorganisms, and age) with regard to their impact on successful treatment of PJI.

## Patients and Methods

We included a consecutive series of 77 patients who had undergone two-stage revision arthroplasty for PJI in the study. The series comprised 35 men and 42 women with a mean age of 64.9 years (range, 31.3-82.4 years). The right knee was affected in 37 cases, the left knee in 40 cases. The median observation period was 24.5 months (range, 6-107 months).

### Diagnosis

Currently, the Musculoskeletal Infection Society (MSIS) criteria is the gold standard for infection diagnosis 10, 11. We assessed the patients' history and clinical findings with regard to previous interventions, symptom duration, as well as general and local comorbidities. We obtained inflammation indices (complete blood count with total and differential leukocyte count, C-reactive protein, fibrinogen, and interleukin-6 [IL-6]), as well as blood cultures in cases of fever or other systemic signs of infection 12. We sent synovial fluid, obtained from joint aspiration, for synovial fluid white blood cell (WBC) count and bacterial cultures. We took standardized radiographs (complete leg, knee in two planes, patella tangential and compared them to previous radiographs, if available, to assess signs of loosening.

Even if the causing bacteria could not be identified preoperatively, patient's history and clinical findings, as well as the presence of elevated inflammation markers and synovial WBC, were sufficient tools in order to obtain security about whether infection was present or not.In cases of inconclusive clinical, laboratory, or bacteriological findings (low-grade infections), we also performed a 99mTc-leukocyte scintigraphy. We diagnosed PJI according to the MSIS criteria. Furthermore, we documented the general health and local wound condition (may contribute to planning intraoperative graft-placement to ensure optimal conditions in the upcoming techniques for PCL reconstruction.

### Surgical technique

#### First-stage procedure

First-stage procedure included the collection of prosthetic specimens (synovia, synovial fluid, and joint capsule) for cultural examination, followed by a thorough debridement and removal of all implants, which we sent for sonication. Subsequently, we placed an antibiotic-loaded bone cement (polymethylmethacrylate [PMMA]) as a static spacer. We used 80-120 grams of PALACOS cement (Heraeus, Germany), which was loaded with 1 gram of vancomycin per 40g of Palacos in most of the cases (94.8%). In some cases, we used different antibiotics, according to the pathogen-specific antibiogram: Antibiotic loading of the spacer comprised vancomycin in almost all cases Tazobactam was applied in 2.6% of cases, while vancomycin/refobacin or teicoplanin and cefuroxime was each used in 1.3% of cases.

#### Spacer retention period

A period of rest between first- and second-stage surgery has been considered necessary in order to ensure treatment success due to infection eradication 13, 14. However, the optimal spacer retention period remains unclear, although several reports have suggested an interval of 6 weeks. In our series, first-stage surgery was followed by an interval of systemic antibiotic administration (usually 6-8 weeks, as recommended in the literature) until the infection was clinically eradicated before the second procedure was performed 3, 12, 13, 14.

#### Second-stage procedure

Infection was considered to be eradicated with the resolution of clinical signs and symptoms, as well as improvement of laboratory infection values. According to our experience, although defining cut-off values has seemed to be challenging in the recent past, a linear decrease followed by steadiness of all infection values within the high-normal to slightly increased range appears to be indicative for treatment success 15.

If those criteria were met, we executed second-stage surgery. We again performed a thorough soft tissue debridement and synovectomy. We removed the joint spacer with care in order to preserve as much bone stock as possible. Finally, after careful debridement of the posterior joint, we performed revision arthroplasty (semi-constrained, stemmed components) in all of the cases in order to achieve stability.

### Postoperative care

The primary goal of the follow-up care was the early detection of re-infection. These follow-up assessments comprised physical examinations, regular laboratory tests (complete blood count, C-reactive protein, and fibrinogen), and imaging procedures, including plain radiographs of the knee in two planes after 6 weeks, 3, 6 and 12 months, and annually thereafter. All patients were followed up every six months for at least two years after second stage surgery.

### Statistical analysis

We entered data into a computerized database and analyzed it. Continuous variables are reported as the median with interquartile range (IQR) or range (minimum-maximum), except for the age, which is described as the mean with standard deviation (SD). Categorical data is displayed as frequencies with percentages in parentheses.

To separate patients with an optimum spacer retention period from patients with a prolonged spacer retention period we used the “maximally selected rank statistic” method 16, calculated with the R package “maxstat” 17. *This method locates the cut-point that maximizes a logrank test statistic as usually applied in connection with a time-to-event analysis. The effect of the spacer retention period on time to reinfection was therefore assessed with a Kaplan-Meier plot with Mantel-Cox logrank test and the hazard ratio for patients with a prolonged spacer retention period of getting a reinfection compared to patients with optimum spacer retention period was assessed with a Cox proportional hazard model adjusted for age and sex.*

*To determine the statistical significance of group differences, we used the Wilcoxon rank-sum test for metric parameters. The relationship between categorical parameters is presented in contingency tables and analyzed with Pearson's chi-squared test. In either case, we determined exact p values; we considered p ≤ 0.05 to be statistically significant.* Computations were performed using the statistical package IBM SPSS Statistics for Windows (Version 24.0, International Business Machines Corporation, Armonk, NY, USA) and R, version 3.3.0.

## Results

### Pre-operative diagnostics and assessment

Clinical manifestations ranged from reduced mobility, impaired range of motion, pain, swelling, and/or redness of the affected region to systemic signs of inflammation or even sepsis. The main clinical symptoms were pain, joint swelling and effusion, overheating, and movement restriction of the affected joint. The median time period between primary joint arthroplasty and onset of the symptoms was 23.6 months (range, 6 months to 336 months).

### Pre-operative laboratory inflammation parameters

The median preoperative C-reactive protein concentration was 61.1 mg/L, the median leukocyte count was 7920 cells/µL, the median fibrinogen concentration was 589 mg/dL, and the median IL-6 concentration was 31.5 pg/dL.

We performed pre-operative joint-aspirations in all patients. An organism could be detected either for pre-operative or both pre- and intra-operative samples in 54 cases (70.1%). In 13 patients (16.9%), bacteria determination was exclusively by implant sonication. The most common identified organisms were staphylococci, followed by streptococci, gram negative rods, and mixed cultures of organisms (Table 1). In 23 (29.8%) of the patients, the infection-causing germ was unidentified.

### Spacer-retention and Second-Stage Management

After first-stage surgery, we continuously evaluated all patients in terms of clinical and laboratory improvements. The median overall spacer retention-period was 10.6 weeks (IQR 8.0 weeks). One additional spacer exchange was necessary in 15 patients (19.7%). Two exchanges were performed in two patients (2.6%) due to persisting signs and symptoms of infection. All patients underwent second-stage surgery.

### Outcome

#### Rate of reinfection and infection-related complications

We observed an 18.7% (n = 14) rate of reinfection until a median time of 19.5 months (IQR 38.9; range, 0-63.9 months). In 11 of these cases, we performed two-stage revision. Two patients underwent transfemoral amputation due to worse local and general conditions. One patient died before completion of the two-stage protocol. Most patients (n=61, 81.3%) stayed infection free until a median follow-up time of 24.6 months (IQR 58.8; range, 6-106.9 months).

#### Timing of reimplantation

**Figure 1:**
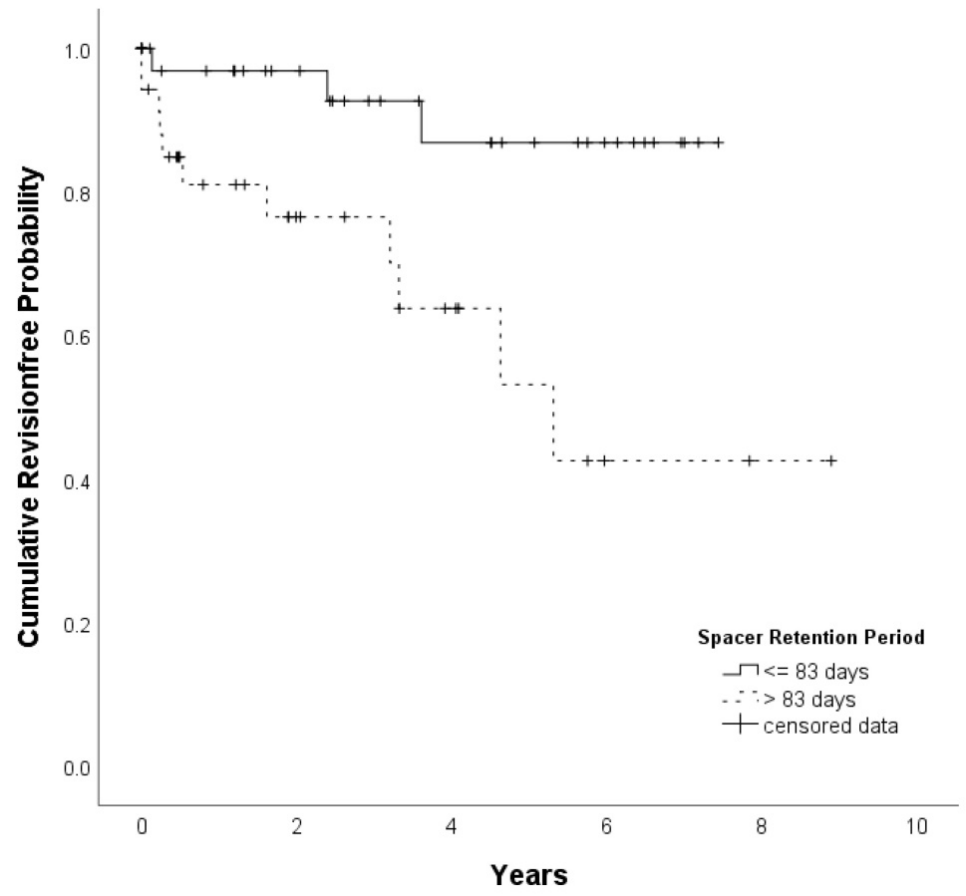
Kaplan-Meier plot of patients with an optimal (less than 83 days) and prolonged (more than 83 days) spacer retention period. The assessed optimal spacer retention period of less than 83 days showed significant better results compared to the group with prolonged intervals (p = .005). 86.9% of all patients with an optimal retention period stayed free from infection by 5 years of follow-up time (vs. 53.1% for patients with prolonged spacer retention periods)

The maximally selected rank statistic method yields a maximum cut-point at 83 days, indicating an optimum spacer retention period of less than 12 weeks. Thirty-nine patients had an optimal and 35 patients a prolonged (more than 12 weeks) spacer retention period. Time to event analysis shows that patients with an optimum spacer retention period have a higher probability of staying free from reinfections than patients with a prolonged spacer retention period (p=.005) (Figure 1).In a proportional hazard modell adjusted for age and sex (Cox regression) we found a hazard ratio of 6.1 (95% CI 1.6 - 22.9) indicating a 6-fold higher risk of getting a reinfection for patients with a prolonged spacer retention period compared to patients with an optimal spacer retention period (p=.007).Patients with a prolonged spacer retention period showed a higher proportion of reinfection (31.4%) compared to patients with an optimal spacer retention period (7.7%) (p = 0.016).

#### Impact of individual parameters

Significant compromising local conditions of the periprosthetic tissue and surrounding skin negatively influenced the outcome. Patients classified as McPherson 10 local extremity grade 3 showed statistically significant more reinfections following two-stage revision surgery for PJI of the knee (50%) as patients with McPherson grade 2 (16.3%) or grade 1 who showed no infections at all (p = 0.005) (Table 2).

A further negative predictor was the number of performed spacer-exchanges between first- and second-stage procedure. An increasing number of spacer exchanges (once, twice, 3 times) was significantly associated with a higher rate of reinfection (10.5%, 40.0% and 100%, respectively) (p = 0.001; Table 3). Neither the patients' age nor gender significantly influenced the outcome regarding reinfection based on the preoperative inflammatory markers.

## Discussion

Although still controversial in the relevant literature, two-stage revision arthroplasty remains the most applied procedure for chronic periprosthetic infections. Antibiotic-loaded cement spacers have become an established method for infection control. The use of high-dose antibiotic-loaded cement as a temporary joint spacer seems to increase the potential for local infection eradication during the time between implant removal and before the second-stage surgery, which aims to restore function and mobility by the use of knee arthroplasty implants once again 3, 18, 19. While two-stage revision is still considered to be the gold standard in the treatment of PJI, especially in the United States, there is growing popularity for the one-stage approach 13, 20, 21. This phenomenon might be due to the fact that one-stage revision, compared to two-stage revision, has the potential to reduce the overall surgical burden on patients as well as healthcare costs 22, 23. Despite these obvious advantages of single-stage revision surgery for PJI, this procedure seems to only be suitable for healthier patients with better bone stock and less virulent organisms only 13.

The goal of surgery must be the complete removal of all infected tissue and implants to eliminate the biofilm and ensure the efficacy of the postoperative antibiotic therapy 5, 24. In our study, we defined treatment success as freedom from signs or symptoms of infection after the end of treatment. Recently, treatment success has been proposed by an expert panel as the microbiological and clinical eradication of infection without relapsed infection, freedom from subsequent surgical intervention for the same infection, and freedom from mortality related to the PJI 24-26.

Next to infection control, which is certainly the primary aim of all treatment protocols, additional goals should be the restoration of function and minimizing infection-related morbidity and mortality 27, 28. Unfortunately, these objectives are not possible for all patients suffering from PJI. Surgical treatment options, although often required, may not be possible or appropriate due to severe comorbidities and a resulting increased perioperative risk. These patients may be best managed conservatively, either by long-term antibiotic suppression, acceptance, or even the creation of a new chronically discharging sinus 5, 24.

Although the technique of two-stage revision surgery seems to be well established for chronic PJI of the knee, different protocols exist among institutions with regard to debridement, spacer choice, and timing of reimplantation, as well as the definition or diagnostic tools used to indicate infection eradication. Failure rates of the two-stage protocol are between 10 and 40% according to recent studies 25, 29-31. The factors associated with failure to ultimately achieve a successful second-stage revision after initial arthroplasty resection are not well understood 31. One suggested reason for treatment failure is a persisting infection, often with the same causative organism(s) 3, 32-35. Another reason for persisting infection following two-stage treatment could be the high incidence of false-negative results of pre- and intraoperative tissue samples with regard to the infection-causing microorganisms. If the biopsy misses the small biofilm population among the involved tissue, there will be a false-negative culture 36. Foregoing antimicrobial therapy or errors during sampling and processing of the specimens may also lead to false results 37-40. Sonication represents a huge advantage in terms of identification of infection-causing microorganisms.

Unfortunately, joint spacers, as they are used to bridge the time between implant removal and replantation, may also develop biofilms 26, 41, 42. Therefore, the timing of second-stage surgery, and thus the spacer retention period, seems to be of high relevance for treatment success. Furthermore, in cases of PJI, clinicians must remember that standard antimicrobial-susceptibility tests cannot be used to reliably predict the outcome. Ideally, the antimicrobial agent should have bactericidal activity against surface-adhering, slow-growing, and biofilm-producing microorganisms. The presence of difficult-to-treat germs as well as undetected infection-causing microorganisms may therefore negatively influence the effect of the antimicrobial treatment 5, 43-46.

In our institutional review, we identified the following as risk factors for failure to achieve an infection-free two-stage revision: a prolonged spacer-retention, McPherson extremity grade 3, and unidentified/difficult-to-treat microorganisms. We observed the best outcome regarding infection-control in patients who had undergone second-stage surgery within 12 weeks of the first-stage surgery. Nearly 90% of these patients stayed free from infection until the final follow-up. Although most authors have suggested spacer-retention periods of 6 weeks, many of our patients exceeded this recommended period of time before completing the two-stage protocol 3-5, 13. This circumstance may be explained by several factors: a bad general condition, which coincides with an increased perioperative risk, persisting infection, and not regularly appearing for follow-up examination within first- and second stage surgery. Furthermore, an increased number of performed spacer-exchanges, as well as a bad local extremity grade, exerted a significant negative impact on the outcome as well in the investigated cohort. We observed an overall reinfection rate of almost 20% following the two-stage exchange protocol for PJI of the knee. This finding is in line with current literature 3, 9, 13, 14, 23, 25, 31. However, due to the retrospective study-design and the variable individual factors which may have also affected the outcome, these findings have to be considered with care. Further prospective studies would be desirable to support our findings.

## Conclusion

We observed the best outcome regarding infection control in patients who had undergone second-stage surgery within 12 weeks after first-stage surgery. Nearly 90% of these patients stayed free from infection until final follow-up. An increased number of performed spacer-exchanges and a bad local extremity grade seemed to have a negative impact on the outcome regarding infection control.

## Figures and Tables

**Table 1:** Number of patients with determined infection-causing microorganisms

**Infection-causing microorganisms**	n	%
E. faecalis	3	3.9
E. faecium	1	1.3
E. faecium, E. faecalis	1	1.3
Group C streptococci	1	1.3
Group G streptococci	1	1.3
MRSA	1	1.3
P. aeruginosa	2	2.6
P. aeruginosa, Bacillus cereus	1	1.3
Pseudomonas aeruginosa, Finegoldia magna	1	1.3
S. aureus	13	16.9
S. aureus, E. faecalis	1	1.3
S. capitis	3	3.9
S. epidermidis	8	10.4
S. hominis	1	1.3
S. hominis, E. faecalis	1	1.3
S. hominis, S. haemolyticus	1	1.3
S. lugdunensis	1	1.3
**Microorganisms detected by sonication (not detected in tissue samples/joint aspirations)**	n	%
P. acnes	1	1.3
P. aeruginosa	1	1.3
S. aureus	4	5.2
S. capitis	2	2.6
S. epidermidis	5	6.5
Undetected microorganisms	23	29.8

The microorganisms were detected either via tissue samples/joint aspiration or the method of sonication. The table shows the frequency (n) and relative proportion of the total patient number (%).

**Table 2:** Reinfection rates following two-stage revision in relation to the local extremity grade

	Reinfection		no Reinfection		Total	
Local extremity grade	n	%	n	%	n	%
**1 (uncompromised)**	0	0.0%	14	100.0%	14	100%
**2 (compromised)**	8	16.3%	41	83.7%	49	100%
**3 (significant compromised)**	6	50.0%	6	50.0%	12	100%
**Total**	14	18.7%	61	81.3%	75	100%

Patients classified as grade 3, according to McPherson et al., showed more reinfections (50%) than grade 2 (16.3%) or grade 1 patients (0%). This was statistically significant (p = 0.005). N defines the number of patients and % the relative proportion of patients for each local extremity grade.

**Table 3:** Reinfection rates following two-stage revision in relation to the number of performed spacer exchanges

	Number of performed spacer exchanges							
	1		2		3		Total	
	n	%	n	%	n	%	n	%
**Reinfection**	6	10.5%	6	40.0%	2	100.0%	14	18.9%
**No Reinfection**	51	89.5%	9	60.0%	0	0.0%	60	81.1%
**Total**	57	100.0%	15	100.0%	2	100.0%	74	100.0%

N describes the number of patients and % the relative proportion of patients who have undergone the respective number of performed spacer exchanges.
